# Biobanked Liver Organoids: A Roadmap for Precision Hepatology

**DOI:** 10.1002/bies.70156

**Published:** 2026-06-29

**Authors:** Elisa J. Cabré, Silvia Cobo‐González, Luis Sánchez Valle, Sonia Martínez, María Del Pilar Ramos‐Álvarez, Yanira Sáez‐Álvarez

**Affiliations:** ^1^ Facultad De Farmacia Universidad San Pablo‐CEU, CEU Universities, Urbanización Montepríncipe Boadilla del Monte España

**Keywords:** biobanking, cryopreservation, FAIR data, governance, Liver organoids, precision hepatology, translational research

## Abstract

Cryopreservation‐enabled workflows decouple tissue acquisition from organoid generation, allowing archived biopsies and surgical explants to be revisited as renewable experimental models. This shift expands access to patient‐specific material, reduces the logistical and batch variability inherent in fresh‐tissue pipelines, and enables retrospective and longitudinal studies anchored in real‐world clinical cohorts. Proof‐of‐concept studies show that liver organoids can be derived from cryopreserved human tissues that retain viability and disease‐relevant phenotypes, but performance remains sensitive to the source material, culture lines, and protocol details. The field remains fragmented, lacking broadly adopted standard operating procedures, shared post‐thaw quality benchmarks, and interoperable data infrastructures linking organoid biobanks to clinical metadata.

In this work, we argue that cryopreservation‐enabled organoid biobanking should be treated as foundational infrastructure for precision hepatology, and we outline a pragmatic roadmap for coordinated implementation over the next decade.

## Graphical abstract

Cryopreservation‐enabled biobanking transforms archived human liver tissue into standardized, quality‐controlled organoid resources. By decoupling organoid generation from the availability of fresh tissue, this infrastructure enables scalable, reproducible, and clinically actionable liver models that support precision hepatology, drug development, and patient‐tailored research.

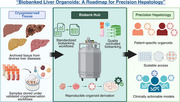

**Abbreviations**
ASCs: Adult Stem/progenitor Cells
CCA: Cholangiocarcinoma
CYP: Cytochrome P450
DILI: Drug‐induced liver injury
DMSO: Dimethyl sulfoxide
FAIR: Findable, accessible, interoperable, and reusable
GDPR: General data protection regulation
GMP: Good manufacturing practices
HCC: Hepatocellular carcinoma
HUB: Hubrecht organoid biobank
iPSC(s): Induced pluripotent stem cell(s)
LICOB: Liver cancer organoid biobank
MASLD: Metabolic dysfunction associated steatotic liver disease
QC: Quality control
SOPs: Standard operating procedures

## 1 Introduction

Chronic liver diseases cause over two million deaths each year, yet therapeutic progress has been slowed by the lack of scalable, physiologically relevant human models [1, 2]. Liver organoids provide a tractable three‐dimensional system that captures key aspects of hepatic architecture and function, supporting applications across disease modeling, toxicology, oncology, and exploratory regenerative strategies [3, 4].

Despite rapid technical maturation, translation has remained constrained by reliance on fresh tissue, variable processing logistics, interlaboratory divergence in media and handling, and limited consensus on the maturity and performance of benchmarks [5, 6, 7, 8, 9, 10, 11, 12, 13].

**FIGURE 1:**
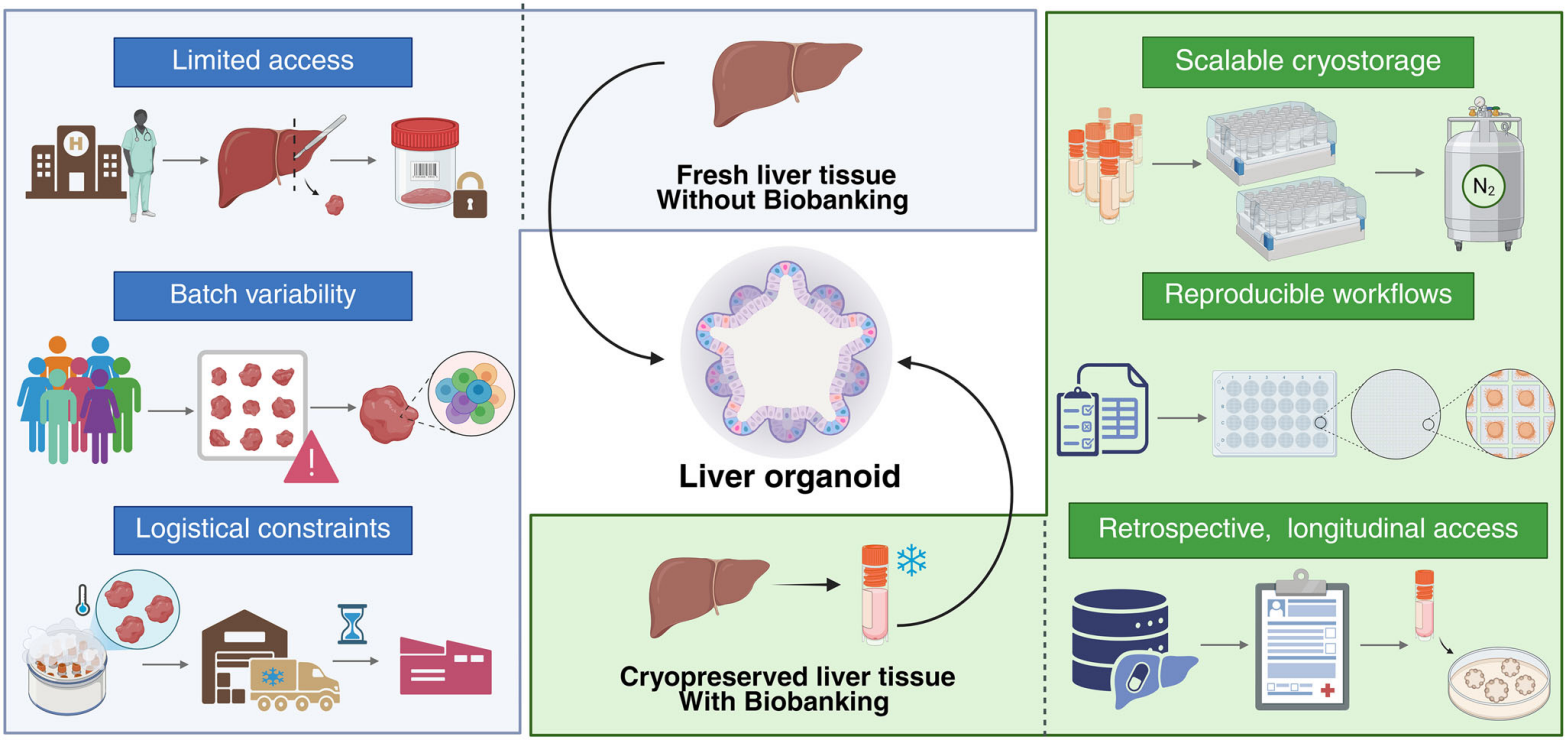
Comparative Scenarios: With versus Without Cryopreservation‐Enabled Biobanking. Side‐by‐side comparison illustrating the challenges of organoid workflows without biobanking — restricted access to fresh tissue, batch‐to‐batch variability, and logistical limitations — contrasted with the benefits of cryopreservation‐enabled biobanks, including scalable cryostorage, standardized, and reproducible derivation pipelines, and the capacity for retrospective and longitudinal analyses — image created with Biorender.

Cryopreservation‐enabled biobanking directly addresses these limitations by decoupling tissue acquisition from the timing of organoid production. Archived liver biopsies and surgical explants can thus be reused to assess organoid reproducibility, enabling retrospective analyses and the creation of large, standardized repositories [5, 8, 14, 15, 16, 17, 18, 19]. By enabling organoid generation from archived liver tissue of diverse clinical origins, biobanks reduce the need for immediate tissue processing, expand access to patient‐specific models [5, 8, 14, 15, 16], and facilitate multicenter collections suitable for systematic comparison in longitudinal and retrospective studies [15, 17] (Figure 1). A roadmap for the next decade must therefore clearly distinguish between establishment capabilities, emerging applications, and aspirational clinical use cases [5, 6, 8, 9, 12, 13].

Nevertheless, the effective implementation of organoid biobanking depends on coordinated advances that extend beyond cryopreservation itself. Consistent adoption of standardized operating procedures (SOPs), Good Manufacturing Practices (GMP) compatible workflows, reproducible quality metrics, and interoperable data infrastructures will be essential to ensure cross‐center comparability and support clinical translation [6, 20, 21, 22, 23, 24]. In parallel, ethical considerations, including long‐term sample governance [25], dynamic consent models [26, 27, 28], and the responsible secondary use of archived material, remain central to the sustainable development of living biobanks [29, 30, 31, 32, 33].

In this perspective, we examine the current challenges facing cryopreservation‐enabled organoid biobanking and propose a roadmap for the coming decade. We focus on how advances in cryobiology and organoid technology [34, 35], the maturation of biobanking infrastructure [7, 30, 36] Findable, accessible, interoperable, and reusable (FAIR) data [14, 36, 37], responsible data handling, and clinical integration can converge to position organoid biobanking as a core complement of precision hepatology [38, 39].

## 2 State of the Art: Liver Organoid Technology and Biobanking

Liver organoid technology has progressed from early proof‐of‐concept systems to platforms that mimic multiple physiologically relevant features of human liver tissue. Organoids derived from either adult stem/progenitor cells (ASCs) or induced pluripotent stem cells (iPSCs) recapitulate core aspects of hepatic organization and function, including apicobasal polarity [40], bile canaliculi networks [41], and clinically relevant metabolic activity [42, 43]. In general, ASCs exhibit more mature hepatic functions. They are therefore particularly used in toxicology and pharmacology studies [44, 45, 46], whereas iPSC‐derived organoids provide an expandable, genetically defined source of material that is particularly suited to modeling developmental and hereditary liver disorders [47, 48, 49, 50, 51] (for better understanding, see Table 1).

**TABLE 1:** Liver organoid classes, typical applications, and biobanking constraints (fit‐for‐overview).

Source Material	Organoid Type/Lineage	Typical Application	Biobanking Constraints (Media/SOP‐Specific)	QC/Identity Metrics	Key Limitations	Reference
Adult liver biopsy/ surgical tissue	Adult epithelial (hepatocyte /ductal/hepatobiliary) organoids	Drug metabolism, DILI/toxicology, epithelial biology	Matrix dependence sensitivity to time‐to‐freeze and digestion; fragmented vs single‐cell freezing impacts recovery; lineage‐supporting media must be preserved across sites	Post‐thaw viability; expansion kinetics; epithelial markers; functional readouts (e.g., CYP activity) aligned to use‐case	Selection bias for expandable clones; incomplete adult maturation in many systems	[44, 45, 46, 61]
Cholangiocyte‐Enriched / Biliary Tissue	Biliary ductal organoids/cholangiocyte‐lineage	Cholangiocyte, bile duct physiology, infection models	Media is heavily signaling‐dependent; identity drift if cues are altered; cryo unit: organoid fragments often require strict passage‐stage control	Lineage markers; barrier/transport readouts: genomic stability checks for long culture	Many underrepresent hepatocyte functions: limited modeling of parenchymal metabolism	[41, 62, 63]
IPSCs (healthy or patient‐derived)	iPSCs‐derived hepatic organoids/ liver buds	Developmental disease, inherited disorders, scalable genetically defined models	The differentiation stage at freeze is critical; xeno‐free transitions affect reproducibility, and batch effects from differentiation workflows dominate unless automated	Differentiation identity panels: maturity metrics: batch QC; Karyotype/ genomic stability	Often, fetal programs are retained; maturation and zonation remain incomplete	[11, 47, 48, 49, 50, 51]
Primary liver cancer tissue	Tumoroids (HCC/CCA) and tumor‐adjacent matched lines	Precision oncology, drug‐response profiling, and resistance mechanisms	High heterogeneity; sampling and necrosis affect take‐rates; tumor‐selective media vary by subtype; cryo timing and tissue quality are major confounders	Histopathology concordance retention; growth kinetics; drug response reproducibility.	Clonal selection and environmental loss may not capture the immune/stromal context	[37, 60, 64]
Co‐culture assemblies /added non‐parenchymal cells	Multicellular stromal /immune ‐augmented organoids	Fibrosis, immune‐liver interactions, microenvironment‐dependent phenotypes	Cryopreservation can differentially affect compartments; reconstitution after that may be required; SOPs must specify composition ratios, and recovery windows	Cell‐type composition QC: single cell or marker panels; functional endpoints matched to the question	Complexity reduces standardization and increases inter‐lab variability	[42, 52, 53]
Archived tissue collections (variable pre‐analyses)	Retrospective organoid derivation (fit‐for‐purpose)	Natural history studies, rare cohorts, pre/post‐treatment comparison	Pre‐analytics are fixed and heterogeneous: acceptance criteria and metadata become critical; higher failure risk must be anticipated and documented	Viability and growth thresholds; identity checks; linkage QC to clinical metadata	Bias towards higher‐quality samples; incomplete clinical metadata can limit interpretability	[14, 65, 66]

A practical roadmap for biobanking should begin by acknowledging the heterogeneity of organoids, which can limit the development of standardized workflows. Liver organoids are not a single, uniform entity: their source material and intended lineage shape isolation strategies, matrix requirements, signaling inputs, expansion kinetics, and the most suitable cryopreservation format (ranging from tissue fragments and cell suspensions to organoid fragments or established lines) (See Table 1). While lineage‐specific media and standard operating procedures remain necessary, and fully universal protocols may not be feasible, important commonalities do exist. Shared infrastructure for handling, quality control, and cryopreservation workflows can significantly streamline implementation. These convergent elements provide a practical foundation for accelerating the development of organoid biobanks, or at least for more standardized, scalable establishment of lineage‐ or tissue‐specific repositories.

Methodological advances have further expanded the physiological scope of liver organoids. The incorporation of non‐parenchymal cell populations, including hepatic stellate cells, endothelial cells, and Kupffer‐like macrophages has enabled modeling of fibrosis, immune‐liver interactions, and aspects of the tumor microenvironment [42, 52, 53] (See Table 1). Despite these improvements, most current liver organoid systems retain fetal transcriptional signatures and exhibit limited metabolic maturation relative to adult liver tissue [54, 55]. To overcome these maturation constraints, emerging platforms increasingly incorporate xeno‐free, chemically defined matrices [56], bioreactor systems that recapitulate oxygen and nutrient gradients [10], and engineered scaffolds that impose spatial cues reminiscent of hepatic zonation [57, 58].

Collectively, these approaches have expanded the experimental applications of liver organoids, supporting disease modeling, pharmacogenomic profiling, and exploratory regenerative strategies [59, 60].

### 2.1 Biobanking as the Next Frontier

Despite the rapid technical maturation of liver organoid systems, their broader translational impact remains limited in the absence of a scalable, standardized biobanking infrastructure [5, 17].

Cryopreservation remains a central bottleneck because it couples physical injury (ice formation, osmotic stress, cryoprotectant toxicity) to biological fragility (matrix dependence, multicellular organization, and post‐thaw stress responses). Slow‐freezing protocols with cryoprotectants such as dimethyl sulfoxide (DMSO) remain widely used for their robustness but can yield variable recovery and disruption of tissue architecture [35, 67, 68]. Consequently, most liver organoid studies have continued to rely on fresh biopsies, which limit scalability and reduce reproducibility across studies.

Proof‐of‐concept studies demonstrate that organoids can be generated from cryopreserved human liver tissue with preserved viability and disease‐relevant phenotypes [8, 15, 16, 34], yet success remains sensitive to pre‐analytic factors (time to freeze, ischemia, tissue quality), cell lineage, and thaw‐recovery practices [14, 69] (See Table 1). If future studies demonstrate that organoids derived from cryopreserved human biopsy material retain viability and functional competence comparable to those generated from fresh biopsies, thereby supporting biobanking as a feasible foundation for large‐scale, standardized organoid repositories.

More advanced approaches, including vitrification, and emerging rewarming (nanowarming) strategies, aim to reduce ice crystallization and improve heat transfer in larger, more complex 3D biological systems [70, 71, 72]. Importantly, while these approaches have shown promising results in the preservation of liver tissue, cut slices, and engineered constructs, direct evidence in standardized liver biobanking workflows remains comparatively limited [72, 73, 74, 75]. These methods are therefore best interpreted as enabling technologies for specific use cases, such as intact tissue fragments, precision‐cut slices, or complex multicellular constructs, rather than as established standards for routine liver organoid biobanking and will require further validation before widespread implementation.

Where evidence is indirect, extrapolation to organoid systems should therefore be made cautiously and within clearly defined experimental boundaries.

Beyond prospective applications, biobanking enables retrospective and longitudinal analyses by linking decades worth of archived liver tissue to contemporary organoid platforms. This capability is especially valuable for low‐prevalence diseases, rare genotypes, and historical clinical cohorts, in which fresh tissue collection is impractical or no longer possible [37, 65]. Archived biopsy collections, for example, can be used to generate organoids for time‐resolved analysis of disease progression or for direct comparisons of pre‐ and post‐treatment states.

To support cross‐center comparability, biobanks need a minimal, fit‐for‐purpose post‐thaw QC panel that can be implemented consistently across centers. At a minimum, this should include four items:

- Viability shortly after thaw
- Early expansion kinetics and organoid‐forming efficiency
- Lineage identity and contamination checks
- Stability assessments proportional to intended use (e.g, genomic stability for long‐term expansion; functional features such as CYP activity for metabolism studies)

It should be noted that current practice varies substantially, complicating comparisons across studies [76, 77, 78].

### 2.2 The Remaining Gap: Standardization

Despite these technical advances, the field lacks harmonized, GMP‐ready SOPS that cover the full biobanking pipeline, including tissue procurement, processing, cryopreservation timing, thawing, and subsequent organoid derivation [51, 79, 80]. Seemingly minor variables, such as time to freeze, medium composition, extracellular matrix formulation, oxygen tension, or passage number, can significantly impact organoid differentiation, metabolic activity, and transcriptomic profiles [5, 10, 12].

Consequently, variability introduced at early stages of the biobanking workflow can propagate through downstream organoid culture and analysis, complicating comparisons across studies and limiting the interpretability of multicenter datasets.

Major initiatives such as the Hubrecht Organoid Biobank (HUB) and Spani's LICOB program have begun to establish shared production pipelines and QC frameworks [18, 19]. Nevertheless, a broadly accepted global consensus on reproducible, clinically aligned standards has yet to be reached.

## 3 Key Translational Barriers to Cryopreservation‐Enabled Liver Organoid Biobanking

The evidence base across liver pathologies remains heterogeneous, with strong signals in specific settings (e.g., tumor organoid biobanks in primary liver cancers) but limited head‐to‐head comparisons of recovery, stability, and phenotypic fidelity across diverse nonmalignant pathologies under matched SOPs [37, 59, 60, 65, 81] (Table 1).

Although organoid derivation from cryopreserved liver tissue has been demonstrated in multiple disease contexts, robust and systematic comparisons of recovery efficiency, bankability, and genomic stability across different liver pathologies (including MASLD, end‐stage cirrhosis, and healthy tissue) under harmonized SOPs remain limited [14, 37, 59, 65]. Consequently, pathology‐specific bankability should presently be considered an evidence gap rather than an established parameter, and a key priority for future comparative and standardization studies.

Despite growing evidence that liver organoids can be reliably generated from cryopreserved tissue, the translation of cryopreservation‐enabled biobanking into a standardized infrastructure remains limited by several interconnected bottlenecks. These challenges include reproducibility, cryopreservation fidelity, workflow standardization, data interoperability, and governance. Addressing these barriers is essential to transform archived liver samples into clinically actionable organoid resources.

### 3.1 Reproducibility Challenges

Reproducibility remains the most significant obstacle for implementing liver organoid biobanking at scale. Variability can arise across multiple stages of the workflow, including tissue procurement [12], enzymatic dissociation [81], extracellular matrix composition [62], growth factor formulations [82], and culture conditions [83]. Even seemingly minor parameters, such as time to freeze after biopsy collection [14], oxygen zonation during culture [10], passage number, or matrix stiffness can substantially influence functional outcomes [13]. This approach acknowledges that recovery potential and stability may differ across disease states, even if these differences have not yet been systematically quantified.

Operationally, biobanks can manage this uncertainty by implementing pathology‐aware acceptance and QC: recording preanalytical variables, stratifying SOP modules by pathology class where feasible, and using minimal performance thresholds that reflect intended downstream use. Practical metrics that are broadly standardizable include post‐thaw viability, early growth kinetics, organoid‐forming efficiency, and basic genomic stability checks (e.g., karyotypes or targeted sequencing for major aberrations) for lines intended for extended expansion (see Table 1 for further information). Pathology‐linked triage criteria, rather than universal expectations, should guide what is banked, re‐banked, or excluded. Inter‐laboratory discrepancies frequently stem from non‐harmonized protocols. However, studies demonstrate that organoids derived from cryopreserved tissue can closely match the viability, genomic integrity, and transcriptomic stability of organoids generated from fresh tissue [8, 15, 16]; reproducibility across centers remains inconsistent. Ongoing efforts, therefore, focus on defining robust and (QC) criteria (post‐thaw viability thresholds, CYP expression stability, and functional benchmarks), to establish equivalence and support regulatory compliance [13].

### 3.2 Cryopreservation Constraints

Standard slow‐freezing protocols using DMSO remain widely adopted because of their robustness, yet they are associated with variable ice formation and inconsistent post‐thaw viability [35, 67]. More advanced approaches, including vitrification and emerging nanowarming strategies, aim to minimize ice crystal formation and achieve more uniform rewarming of complex three‐dimensional tissues [35, 72, 84]. However, these methods require specialized equipment and have not yet been broadly implemented across biobanks.

Furthermore, standardized post‐thaw quality metrics are still lacking. Criteria such as cell viability, organoid‐forming efficiency, hepatic marker expression, and metabolic activity vary substantially between laboratories, complicating cross‐study comparisons [76, 77]. Establishing precise and reproducible performance thresholds will be essential for demonstrating equivalence between organoids derived from fresh and frozen samples and for supporting GMP‐ready workflows.

### 3.3 Scaling Workflows and Infrastructure

Transitioning from individual laboratories to large‐scale biobanking requires uniform standards for tissue handling, annotation, freezing, storage, and shipment. At present, however, current practices vary widely across institutions [5, 30, 63, 85].

International initiatives have begun addressing these gaps. The HUB, for example has pioneered standardized production pipelines for organoid lines [17, 18], while Spain's LICOB initiative integrates organoid generation into a structured framework for drug discovery and clinical research [19]. Nevertheless, a globally harmonized, GMP‐ready framework for cryopreserved organoid repositories spanning research, preclinical, and clinical settings remains lacking.

Infrastructure disparities further exacerbate these challenges. Cryogenic facilities, redundant storage capacity, QC testing, and trained personnel remain concentrated in high‐income regions, raising concerns about the emergence of a “biobank divide” that could limit global equity in access to organoid‐based resources [27, 29].

### 3.4 Standardization, Interoperability, and Governance: Making Organoids Usable at Scale

Scaling from individual laboratories to networked biobanking requires more than freezing protocols. The high objective is reproducible ‘downstream behaviors’ across sites: comparable recovery, identity, and performance under shared QC criteria. Initiatives such as HUB and LICOB demonstrate the feasibility of coordinated pipelines [18, 19], yet a globally harmonized, GMP‐aligned framework spanning research to early translational settings remain incomplete.

Linking organoid biobanks with clinical records remains a significant obstacle. Interoperability is constrained by heterogeneous clinical coding systems, jurisdiction‐specific privacy regimes (including GDPR), and uneven adoption of minimum metadata standards [85, 86, 87, 88, 89]. FAIR principles are necessary but not sufficient: biobanks also require traceable sample identity, versioned SOPs, and audit‐ready provenance linking a physical vial to its derived organoid line(s), QC outcomes, and analytical outputs. Federated and privacy‐preserving approaches can support cross‐site analytics without forcing raw data centralization, but they demand shared ontologies and governance agreements before they become operational defaults [87, 88, 89].

Without clinically annotated organoid libraries, the development of patient‐matched models for personalized hepatology remains limited. Synchronizing ‘digital twins’ [90] with physical samples should be treated as a lifecycle problem. At minimum, digital representations must encode sample identity, processing history, cryo conditions, passage number, QC result, and derived data products, with explicit versioning whenever an organoid line is expanded, genetically modified, or altered.

Cross‐jurisdictional operation will likely require stratified access control, dynamic consent pathways that remain auditable over time [26, 27, 28], and clear material transfer and end‐benefit‐sharing terms that prevent ‘biobank dividing dynamics' in which capacity and access remain concentrated in high‐income regions [27, 29].

### 3.5 Ethical and Governance Barriers

The biobanking of organoid‐forming tissue introduces complex ethical considerations that differ substantially from those of traditional biobanks. Donors should be informed that their samples may yield organoid lines that self‐renew indefinitely, potentially for future research, commercial applications, or regenerative therapies [31, 32, 33].

Dynamic consent models, which allow donors to adapt their permissions over time, represent a promising alternative to static consent, yet remain limited in implementation [27, 28, 31, 32, 91, 92]. Additional challenges arise from the secondary use of archived diagnostic or surgical tissue, in which original consent may not cover organoid derivation or long‐term storage [69]. Clear governance frameworks must balance innovation with respect to autonomy, transparency, and equitable access [26, 92].

### 3.6 Clinical Integration and Translational Positioning Within the Roadmap

The translational value of cryopreserved organoid biobanks will depend on clinically annotated libraries that enable patient/matched testing, retrospective cohort analyses, and multi‐center benchmarking. In the near term, the most realistic touchpoints are ex vivo applications that clearly define endpoints (e.g., toxicity signals, pathway engagement, or drug response phenotypes) rather than claims of routine clinical decision support.

### 3.7 Cell‐Based Therapy and Organoid‐Based Modeling sit on Different Translational Tracks

Regenerative approaches require GMP‐grade manufacturing, stringent release criteria, and long‐term follow‐up with clinically meaningful endpoints anchored to engraftment, function, and adverse events [20, 93, 94]. By contrast, organoid modeling aims to generate actionable functional readouts (drug response, toxicity, phenotype stratification) without implantation, which can reduce regulatory burden and shorten iteration cycles but also introduces risks: limited maturation, selection bias during culture, and uncertain correspondence between *ex vivo* endpoints and patient outcomes. Scalability also diverges; modeling can often tolerate panel‐based standardization and federated analysis, whereas therapy requires tightly controlled product identity and consistency.

In roadmap framing, organoid biobanks are best positioned as near‐ to mid‐term infrastructure for discovery, stratification, and trial enablement, while providing a structured substrate for longer‐horizon regenerative programs rather than substituting for them.

## 4 Roadmap for the Next Decade (2025‐2035)

The coming decade is likely to be decisive for establishing cryopreservation‐enabled organoid biobanking as a foundational infrastructure for precision hepatology [1, 2, 3, 95]. While proof‐of‐concept studies have demonstrated that high‐quality liver organoids can be generated from cryopreserved tissue [8, 15, 16]. The field now faces the challenge of translating this capability into a coordinated and interoperable global system. Progress from fragmented laboratory practices towards integrated biobanking infrastructures will require advances in cryobiology, standardization, data integration, and ethical governance [5, 7, 17, 30, 35]. Importantly, these developments must proceed in parallel rather than sequentially, as progress in each domain is tightly interdependent.

**FIGURE 2:**
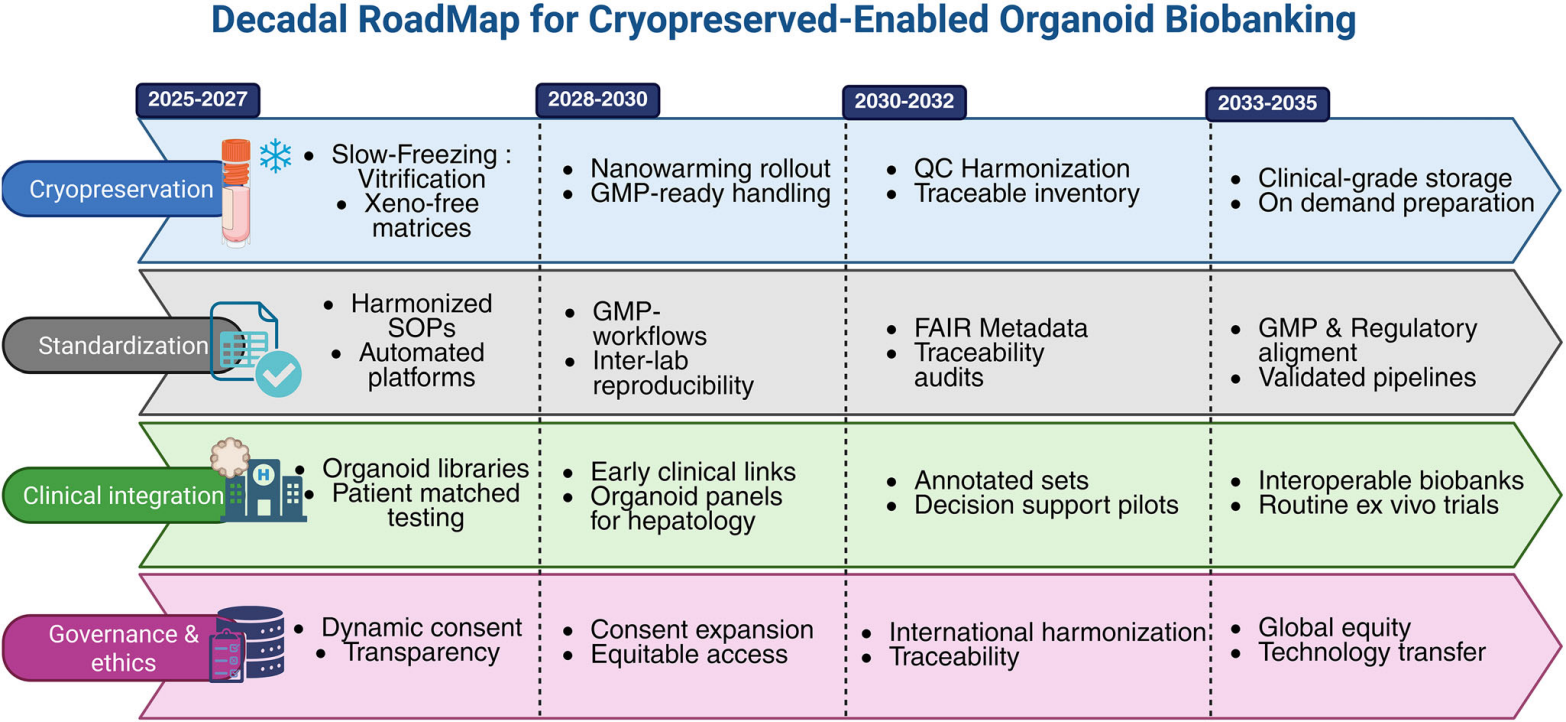
Decadal Roadmap (2025–2035) for Cryopreservation‐Enabled Liver Organoid Biobanking: A roadmap illustrating the anticipated evolution of liver organoid biobanking over the next decade. Early milestones (2025–2027) focus on harmonizing SOPs, defining post‐thaw quality‐control metrics, and implementing automated workflows. Mid‐term progress (2028–2030) includes vitrification and nanowarming, GMP‐ready platforms, and linkage to clinical datasets. Later phases (2030–2032) emphasize alignment with international protocols, expanded dynamic consent, and FAIR‐compliant traceability. By 2033–2035, interoperable clinical biobanks are expected to enable organoids‐on‐demand for ex vivo testing and early translational applications — image created with BioRender.

In the early phase of this decade (2025‐2027), efforts are expected to focus on establishing robust and widely accepted standard operating procedures for tissue freezing, thawing, and organoid derivation. Current protocols vary substantially across laboratories, and the absence of shared quality‐control metrics, such as minimal post‐thaw viability [4, 5, 6, 7, 30, 96]. Expansion rates, metabolic benchmarks, and genomic integrity thresholds continue to limit reproducibility [29, 65, 79, 97]. A key early milestone will therefore be the publication and adoption of harmonized SOPs, accompanied by inter‐laboratory comparisons demonstrating that organoids derived from Figure 2. Decadal Roadmap (2025–2035) for Cryopreservation‐Enabled Liver Organoid Biobanking: A roadmap illustrating the anticipated evolution of liver organoid biobanking over the next decade. Early milestones (2025–2027) focus on harmonising SOPs, defining post‐thaw quality‐control metrics, and implementing automated workflows. Mid‐term progress (2028–2030) includes vitrification and nanowarming, GMP‐ready platforms, and linkage to clinical datasets. Later phases (2030–2032) emphasize alignment with international protocols, expanded dynamic consent, and FAIR‐compliant traceability. By 2033–2035, interoperable clinical biobanks are expected to enable organoids‐on‐demand for ex vivo testing and early translational applications (image created with BioRender)Figure 2: Decadal Roadmap (2025–2035) for Cryopreservation‐Enabled Liver Organoid Biobanking: A roadmap illustrating the anticipated evolution of liver organoid biobanking over the next decade. Early milestones (2025–2027) focus on harmonising SOPs, defining post‐thaw quality‐control metrics, and implementing automated workflows. Mid‐term progress (2028–2030) includes vitrification and nanowarming, GMP‐ready platforms, and linkage to clinical datasets. Later phases (2030–2032) emphasize alignment with international protocols, expanded dynamic consent, and FAIR‐compliant traceability. By 2033–2035, interoperable clinical biobanks are expected to enable organoids‐on‐demand for ex vivo testing and early translational applications (image created with BioRender) frozen samples reproducibly recapitulate the biological traits of those derived from fresh tissue across centers [8, 14, 15, 16].

This period is also likely to include initial piloting of vitrification and emerging rewarming strategies, such as nanowarming, particularly for more delicate or complex samples [67, 70, 72, 78, 84, 98]. In parallel, the progressive adoption of xeno‐free matrices and chemically defined media that align with regulatory expectations is expected to accelerate [20, 22, 23, 24, 56, 62, 82]

As these foundations solidify, the mid‐decade period (2028‐2032) is expected to shift toward scaling and harmonizing biobanking infrastructures. Regional and national biobanks often build initiatives such as HUB or LICOB [18, 19, 30, 35, 99], are likely to implement shared frameworks for sample annotation, batch tracking, viability assessment, and functional testing. Increasing the automation of critical steps, including controlled rate freezing, thawing, aliquoting, and QC assays, should further improve reproducibility and reduce operator‐dependent variability [57, 78].

The consolidation of interoperable biobanks during this phase will also facilitate multicenter studies designed to evaluate pharmacological responses, disease modeling, robustness, and inter‐donor variability using standardized organoid lines [37, 38, 39, 60, 64, 65, 100]. Significantly, the translational value of these efforts will increasingly depend on data infrastructures. The adoption of FAIR principles [86, 89], together with the development of federated and privacy‐preserving architectures, will enable organoid resources to be linked to clinical and genomic metadata [60, 65, 85, 87, 88, 89], thereby achieving contextual integration that is difficult to achieve with fresh tissue‐based models.

By the end of the decade (2033‐2035), a mature network of living liver biobanks could begin to influence selected areas of clinical practice. If early pilot efforts are successful, organoids derived from archived or prospectively collected cryopreserved samples may serve as individualized functional avatars to support therapeutic decision‐making in specific indications [64, 81, 100, 101, 102, 103]. In parallel, retrospective derivation of organoids from well‐annotated clinical cohorts could yield new insights into disease trajectories, drug‐induced liver injury, and rare metabolic phenotypes [81, 104].

At the same time, continued integration of non‐parenchymal cells and advances in organoid maturation strategies [11, 40, 42, 52, 53, 54] may position biobanked organoids for early translational applications, including ex vivo drug testing and preparatory steps toward GMP‐compatible regenerative approaches [20, 22, 23, 24, 37, 43, 105]. The long‐term sustainability of this ecosystem, however, will depend on the establishment of globally coordinated governance structures that ensure equitable access, clearly defined consent pathways, and transparent benefit‐sharing models [25, 26, 27, 28, 29, 31, 32, 33, 92].

Taken together, this decade‐long roadmap describes a transition from technical feasibility to scalable, standardized, and ethically grounded implementation (Figure 2). Achieving this shift will require sustained collaboration among cryobiologists, organoid engineers, clinicians, ethicists, data scientists, and regulatory bodies. If these efforts converge, cryopreservation‐enabled biobanking has the potential to reshape liver research and clinical care by providing a renewable, reproducible, and clinically anchored organoid resource over the long term.

## 5 Future Opportunities for Precision Hepatology

**FIGURE 3:**
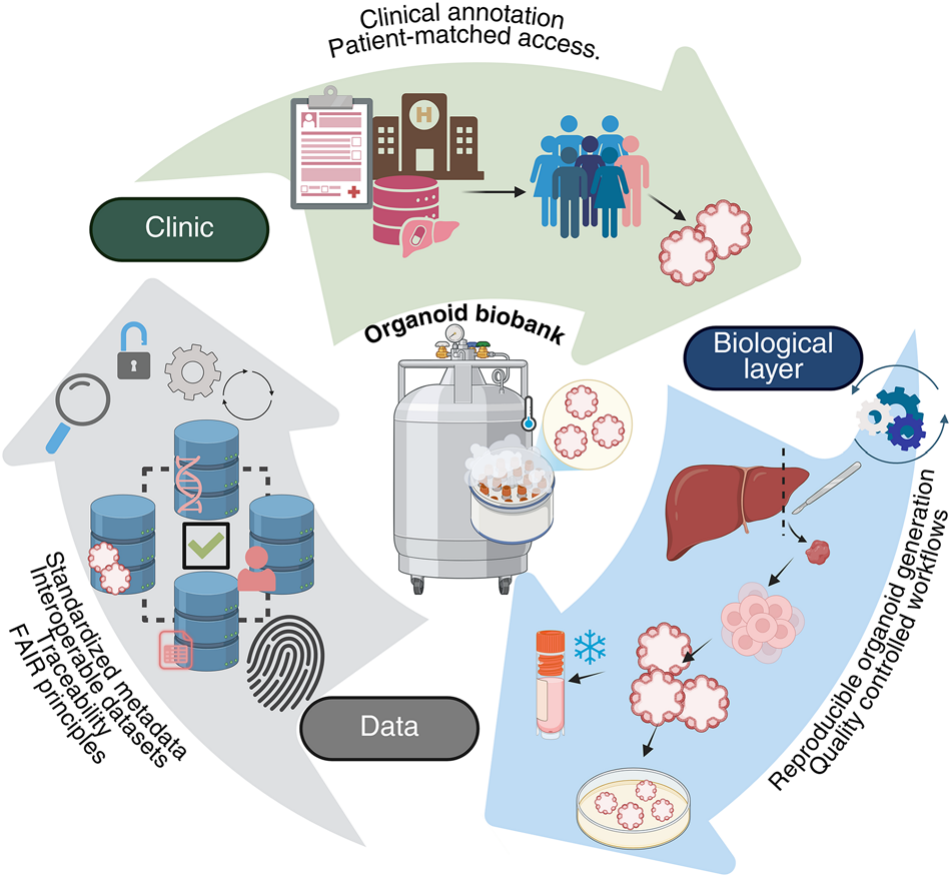
Integrated Ecosystem Linking Biobanks, Clinical Data, and Organoid Platforms: Conceptual overview of an integrated biobanking framework that connects clinical annotation, standardized data systems, and the biological generation of liver‑derived organoids. The figure illustrates how patient‑matched information, harmonized metadata, and controlled organoid workflows converge to support a coherent ecosystem in which clinical inputs and experimental processes continually reinforce one another — image created with BioRender.

Cryopreservation‐enabled organoid biobanking offers an alternative opportunity to move hepatology beyond reactive care toward more predictive, stratified, and individualized approaches. Beyond improvements in standardization and scalability, such biobanks enable scientific and clinical capabilities that are difficult to achieve using fresh‐tissue workflows alone. (Figure 3).

### 5.1 Building Comprehensive Reference Atlases of Human Liver Diversity

Large and ethnically diverse biobanks can support the development of organoid‐based reference atlases that capture inter‐individual variation in hepatic metabolism, drug response, and disease susceptibility [42, 60, 99, 106, 107, 108]. These atlases could complement single‐cell and spatial transcriptomic datasets by providing a renewable and experimentally tractable platform for functional interrogation [51, 107, 108]. Organoid libraries generated from cryopreserved samples, particularly when linked to donor genotypes, may become valuable tools for investigating population‐level hepatic diversity and rare metabolic phenotypes [59, 106].

### 5.2 Transforming Drug Development Through Reproducible Human Models

Biobanked liver organoids can function as standardized human testbeds for early‐stage pharmacology, toxicity screening, and mechanism‐of‐action studies. Their ability to recapitulate clinically relevant cytochrome P450 activity and bile canaliculi architecture positions them as higher‐fidelity alternatives to traditional hepatocyte cultures [45, 101, 109]. As organoid QC metrics become increasingly harmonized [61, 63], pharmaceutical development pipelines could incorporate organoid‐based assays for cross‐cohort toxicity comparisons, stratified drug testing, and the prioritization of candidate compounds before clinical evaluation [64, 65, 109]. The ability to regenerate equivalent organoid lines across laboratories further facilitates multicenter validation efforts.

### 5.3 Enabling Retrospective and Longitudinal Clinical Research

Unlike fresh tissue, cryopreserved samples can be accessed years or decades after collection. This capacity creates new opportunities to generate retrospective organoids from clinically annotated cohorts [66]. Such approaches may accelerate research in low‐prevalence diseases, rare genotypes, pediatric disorders, and drug‐induced liver injury (DILI), where fresh material is inherently limited [3, 95]. Retrospective organoid derivation also enables time‐resolved analyses, including comparisons of organoids generated from samples collected before and after disease progression or therapeutic exposure [66, 110].

### 5.4 Patient's Avatars for Therapeutic Decision

Patient‐matched liver organoids derived from cryopreserved biopsies could serve as functional avatars to inform individualized therapeutic decision‐making [39, 96, 100, 102, 111]. Although clinical validation is still ongoing, proof‐of‐concept studies in other organ systems suggest that *ex vivo* drug‐response profiles correlate with clinical outcomes. In this context, biobanked liver organoids may support personalized strategies for metabolic disorders, cholestatic disease, viral hepatitis, and early‐stage liver cancer, especially when combined with pharmacogenomic data [100, 102, 112, 113, 114]

### 5.5 Foundations for Regenerative Hepatology

Although liver organoids are not yet suitable for direct transplantation, cryopreserved tissue biobanks can support systematic investigation of organoid‐based regenerative strategies [1, 115]. Advances in co‐culture systems and the incorporation of non‐parenchymal liver cell types may enable the generation of organoids with enhanced maturation and immunological competence [2, 3, 94]. As xeno‐free matrices and GMP‐compatible workflows continue to mature, biobanked organoids could ultimately contribute to future cell‐based therapies or to the production of extracellular vesicles, secretomes, or engineered tissue constructs [56, 72, 116].

### 5.6 Enabling Global Equity in Liver Research and Precision Care

By decoupling organoid generation from immediate access to fresh tissue, cryopreservation lowers barriers for centers with limited surgical throughput or specialized culturing facilities [27, 29, 117]. When combined with dynamic consent models, FAIR data standards, and shared QC frameworks, organoid biobanking can democratize access to high‐quality hepatology models across geographic and socioeconomic boundaries [26, 28, 86, 112]. Such expansion is critical for developing therapies relevant to diverse populations, particularly for metabolic disease and viral hepatitis, whose global burdens are unevenly distributed.

## 6 Conclusion and Outlook

Cryopreservation‐enabled organoid biobanking is emerging as an enabling infrastructure for hepatology by decoupling organoid generation from fresh tissue constraints and enabling retrospective access to clinically anchored samples. Proof‐of‐concept studies support the feasibility of deriving liver organoids from cryopreserved tissue while preserving viability and disease‐relevant phenotypes [8, 15, 16, 34]. However, performance remains protocol‐ and linkage‐dependent, with broadly harmonized benchmarks still lacking.

Evidence demonstrates that liver organoids derived from cryopreserved samples retain genomic integrity, functional maturity, and disease‐relevant phenotypes comparable to those generated from fresh tissue [8, 9, 15, 16, 34, 69]. This capability effectively converts archived biopsies and surgical remnants into renewable platforms for modeling hepatic physiology and pathology at scale.

The coming decade will determine whether this technical feasibility can mature into a globally harmonized and sustainable system. Progress will depend on the convergence of three key pillars.

I) Standardized cryopreservation workflows with fit‐for‐purpose post‐thaw QC thresholds to ensure reproducibility across centers [78, 79, 96].
II)Interoperable, FAIR‐aligned data systems with auditable origin and privacy‐preserving linkage to clinical metadata [85, 86, 87, 88, 89].
III)Governance frameworks that support dynamic consent, equitable access, and transparent benefit‐sharing [25, 26, 27, 28, 29, 31, 32, 33].

If these three conditions are met, living liver organoid biobanks can shift the field towards more reproducible modeling and better translational studies, while providing a disciplinary substrate for longer‐horizon regenerative ambitions. Ultimately, biobanks represent far more than repositories of biological material. They constitute dynamic infrastructures capable of linking past, present, and future clinical data and biological insight. As these systems mature, they may help drive a transition shift toward predictive, equitable, and personalized approaches to liver medicine.

**Outstanding questions box**

What tier of harmonization (minimum metadata and core QC vs lineage‐specific SOP modules) is sufficient to support multi‐center reproducibility?

Which post‐thaw QC metrics best predict long‐term functional performance for specific use cases (metabolism, fibrosis, oncology, cholangiopathies.)?

How can vitrification, nano‐warming, and other emerging cryobiology technologies be standardized and validated for routine use in human liver tissue preservation?

What metadata frameworks and FAIR data structures are necessary to enable secure, interoperable linkage between organoid biobanks, clinical records, and genomic datasets?

How can dynamic consent be implemented at scale with clear versioning, withdrawal, handling, and cross‐site access control?

Where do *ex vivo* organoids endpoints reliably correlate with clinical outcomes, and where are they likely to fail?

To what extent can patient‐derived organoids serve as functional avatars for predicting drug response, hepatotoxicity, or disease progression in real‐world clinical settings?

What are the realistic go/no‐go criteria for transitioning organoid resources toward GMP–aligned pipelines for regenerative applications?

How can capacity building be translated into practice to prevent inequalities in biobanking and support fair global access and benefit sharing?

## Author Contributions

**Yanira Sáez‐Álvarez, Elisa J. Cabré**: Conceptualization. **Silvia Cobo‐González**: Literature review. **Yanira Sáez‐Álvarez, Elisa J.Cabré**: Writing – original draft. **María Del Pilar Ramos‐Álvarez, Sonia Martínez Barrios, Yanira Sáez‐Álvarez, Elisa J. Cabré**: Writing – review and editing. **Silvia Cobo‐González, Yanira Sáez‐Álvarez**: Visualization.

## Funding

This review received no specific grant from any funding agency, whether commercial or not‐for‐profit.

## Conflicts of Interest

The authors declare no competing interests.

## Data Availability

All information discussed in this review is derived from published literature cited in the reference list. No new data was generated.

## References

[1] Y. Kim , M. Kang , M. G. Mamo , M. Adisasmita , M. Huch , and D. Choi , “Liver Organoids: Current Advances and Future Applications for Hepatology,” Clinical and Molecular Hepatology 31 (2025): S327–S348, 10.3350/cmh.2024.1040. 39722609 PMC11925438

[2] D. Gong , J. Mo , and M. Zhai , “Advances, Challenges and Future Applications of Liver Organoids in Experimental Regenerative medicine,” Frontiers in Medicine 11 (2025): 1521851, 10.3389/fmed.2024.1521851. 39927267 PMC11804114

[3] S. Nuciforo and M. H. Heim , “Organoids to Model Liver Disease,” JHEP Reports 3, no. 1 (2021): 100198, 10.1016/j.jhepr.2020.100198. 33241206 PMC7672322

[4] C. Caiazza , S. Parisi , and M. Caiazzo , “Liver Organoids: Updates on Disease Modeling and Biomedical Applications,” Biology 10, no. 9 (2021): 835, 10.3390/biology10090835. 34571712 PMC8470787

[5] W. Huang , Z. Xu , S. Li , J. Zhou , and B. Zhao , “Living Biobanks of Organoids: Valuable Resource for Translational Research,” Biopreservation and Biobanking 22, no. 6 (2024): 543–549, 10.1089/bio.2023.0142. 38959173 PMC11656124

[6] S.‐J. Ahn , S. Lee , D. Kwon , et al., “Essential Guidelines for Manufacturing and Application of Organoids,” International Journal of Stem Cells 17, no. 2 (2024): 102–112, 10.15283/ijsc24047. 38764240 PMC11170116

[7] R. Yang , Y. Qi , X. Zhang , H. Gao , and Y. Yu , “Living Biobank: Standardization of Organoid Construction and Challenges,” Chinese Medical Journal 137, no. 24 (2024): 3050–3060, 10.1097/CM9.0000000000003414. 39663560 PMC11706585

[8] Y.‐H. Tsai , M. Czerwinski , A. Wu , et al., “A Method for Cryogenic Preservation of Human Biopsy Specimens and Subsequent Organoid Culture,” Cellular and Molecular Gastroenterology and Hepatology 6, no. 2 (2018): 218–222, 10.1016/j.jcmgh.2018.04.008. 30105282 PMC6085494

[9] T. Takebe , K. Sekine , M. Kimura , et al., “Massive and Reproducible Production of Liver Buds Entirely From Human Pluripotent Stem Cells,” Cell Reports 21 (2017): 2661–2670, 10.1016/j.celrep.2017.11.005. 29212014

[10] T. Utami , M. Danoy , R. R. Khadim , et al., “A Highly Efficient Cell Culture Method Using Oxygen‐permeable PDMS‐based Honeycomb Microwells Produces Functional Liver Organoids From human Induced Pluripotent Stem Cell‐derived Carboxypeptidase M Liver Progenitor Cells,” Biotechnology and Bioengineering 121, no. 4 (2024): 1177–1189, 10.1002/bit.28640. 38184815

[11] Y. Weng , S. Han , M. T. Sekyi , T. Su , A. N. Mattis , and T. T. Chang , “Self‐Assembled Matrigel‐Free iPSC‐Derived Liver Organoids Demonstrate Wide‐Ranging Highly Differentiated Liver Functions,” Stem Cells 41, no. 2 (2023): 126–139, 10.1093/stmcls/sxac090. 36573434 PMC9982071

[12] L. Guo , C. Li , and W. Gong , “Toward Reproducible Tumor Organoid Culture: Focusing on Primary Liver cancer,” Frontiers in Immunology 15 (2024): 1290504, 10.3389/fimmu.2024.1290504. 38571961 PMC10987700

[13] S. Shrestha , M. G. Vanga , C. Jonnadula , P. Acharya , M. Lee , and M.‐Y. Lee , “Reproducible, Scale‐Up Production of Human Liver Organoids (HLOs) on a Pillar Plate Platform via Microarray 3D Bioprinting,” (2025), 10.1007/7651_2024_603. 39821806

[14] C. Goossens , V. Tambay , V.‐A. Raymond , L. Rousseau , S. Turcotte , and M. Bilodeau , “Impact of the Delay in Cryopreservation Timing During Biobanking Procedures on human Liver Tissue Metabolomics,” PLoS ONE 19, no. 6 (2024): 0304405, 10.1371/journal.pone.0304405. PMC11164386 38857235

[15] A. He , S. Powell , M. Kyle , et al., “Cryopreservation of Viable Human Tissues: Renewable Resource for Viable Tissue, Cell Lines, and Organoid Development,” Biopreservation and Biobanking 18, no. 3 (2020): 222–227, 10.1089/bio.2019.0062. 32302515 PMC7310214

[16] L. Nevi , V. Cardinale , G. Carpino , et al., “Cryopreservation Protocol for human Biliary Tree Stem/Progenitors, Hepatic and Pancreatic Precursors,” Scientific Reports 7, no. 1 (2017): 6080, 10.1038/s41598-017-05858-0. 28729654 PMC5519713

[17] F. J. Di Paola , G. Calafato , P. P. Piccaluga , G. Tallini , and K. J. Rhoden , “Patient‐Derived Organoid Biobanks for Translational Research and Precision Medicine: Challenges and Future Perspectives,” Journal of Personalized Medicine 15, no. 8 (2025): 394, 10.3390/jpm15080394. 40863456 PMC12387782

[18] J. Mullenders , A. Buijs , L.‐E. Fielmich , et al., “Abstract 126: HUB Organoids: Bringing the “Patient in the Lab” for Preclinical and Clinical Development,” Cancer Research 81, no. 13_Supplement (2021): 126–126, 10.1158/1538-7445.AM2021-126.

[19] H. Jin , Z. Sun , and R. Bernards , “LICOB: A Powerful Organoid Platform for Drug Discovery,” Cell Research 34, no. 1 (2023): 11–12, 10.1038/s41422-023-00878-0. PMC10770056 37730938

[20] D. J. Beetler , D. N. Di Florio , E. W. Law , et al., “The Evolving Regulatory Landscape in Regenerative Medicine,” Molecular Aspects of Medicine 91 (2023): 101138, 10.1016/j.mam.2022.101138. 36050142 PMC10162454

[21] C. Corrò , L. Novellasdemunt , and V. S. W. Li , “A Brief History of Organoids,” American Journal of Physiology‐Cell Physiology 319, no. 1 (2020): C151–C165, 10.1152/ajpcell.00120.2020. 32459504 PMC7468890

[22] L. Zhou , J. Huang , C. Li , et al., “Organoids and Organs‐on‐chips: Recent Advances, Applications in Drug Development, and Regulatory Challenges,” Med 6, no. 4 (2025): 100667, 10.1016/j.medj.2025.100667. 40220744

[23] U.S. Food and Drug Administration (FDA). (2024). Potential Approaches to Drive Future Integration of New Alternative Methods for Regulatory Decision‐Making.

[24] European Medicines Agency . (2024). Regulatory acceptance of new approach methodologies (NAMs) to reduce animal use testing.

[25] F. Gille , E. Vayena , and A. Blasimme , “Future‐proofing Biobanks′ governance,” European Journal of Human Genetics 28, no. 8 (2020): 989–996, 10.1038/s41431-020-0646-4. 32424324 PMC7468350

[26] R. Thompson , K. Lyle , G. Samuel , et al., “The Research Relationship: Participant Perspectives on Consent in Biobanking,” BMC Medical Ethics 26, no. 1 (2025): 47, 10.1186/s12910-025-01199-0. 40221732 PMC11992699

[27] M. Prictor , H. J. A. Teare , and J. Kaye , “Equitable Participation in Biobanks: The Risks and Benefits of a “Dynamic Consent” Approach,” Frontiers in Public Health 6 (2018): 410631, 10.3389/fpubh.2018.00253. PMC6133951 30234093

[28] R. Isasi , H. B. Bentzen , M. Fabbri , et al., “Dynamic Governance: A New Era for Consent for Stem Cell Research,” Stem Cell Reports 19, no. 9 (2024): 1233–1241, 10.1016/j.stemcr.2024.07.006. 39151430 PMC11411296

[29] Y. S. Lee , N. L. B. Garrido , G. Lord , Z. A. Maggio , and B. B. Khomtchouk , “Ethical Considerations for Biobanks Serving Underrepresented Populations,” Bioethics 39, no. 3 (2025): 240–249, 10.1111/bioe.13381. 39659164 PMC11831713

[30] L. Annaratone , G. De Palma , G. Bonizzi , et al., “Basic Principles of Biobanking: From Biological Samples to Precision Medicine for Patients,” Virchows Archiv 479, no. 2 (2021): 233–246, 10.1007/s00428-021-03151-0. 34255145 PMC8275637

[31] M. A. Lensink , K. R. Jongsma , S. N. Boers , J. J. Noordhoek , J. M. Beekman , and A. L. Bredenoord , “Responsible Use of Organoids in Precision Medicine: The Need for Active Participant Involvement,” Development 147, no. 7 (2020): 177972, 10.1242/dev.177972. 32253255

[32] J. Lewis and S. Holm , “Organoid Biobanking, Autonomy and the Limits of Consent,” Bioethics 36, no. 7 (2022): 742–756, 10.1111/bioe.13047. 35531912 PMC9542633

[33] S. N. Boers , J. J. van Delden , H. Clevers , and A. L. Bredenoord , “Organoid Biobanking: Identifying the Ethics,” The EMBO Reports 17, no. 7 (2016): 938–941, 10.15252/embr.201642613. 27296278 PMC4931554

[34] H. Han , T. Zhan , N. Guo , M. Cui , and Y. Xu , “Cryopreservation of Organoids: Strategies, Innovation, and Future Prospects,” Biotechnology Journal 19, no. 2 (2024): e2300543, 10.1002/biot.202300543. 38403430

[35] I. V. Khaydukova , V. M. Ivannikova , D. A. Zhidkov , et al., “Current State and Challenges of Tissue and Organ Cryopreservation in Biobanking,” International Journal of Molecular Sciences 25, no. 20 (2024): 11124, 10.3390/ijms252011124. 39456905 PMC11508709

[36] X. Xie , X. Li , and W. Song , “Tumor Organoid Biobank‐new Platform for Medical Research,” Scientific Reports 13, no. 1 (2023): 1819, 10.1038/s41598-023-29065-2. 36725963 PMC9892604

[37] A. A. Qureshi , C. J. Wehrle , S. Ferreira‐Gonzalez , et al., “Tumor Organoids for Primary Liver Cancers: A Systematic Review of Current Applications in Diagnostics, Disease Modeling, and Drug Screening,” JHEP Reports 6, no. 12 (2024): 101164, 10.1016/j.jhepr.2024.101164. 39583095 PMC11584567

[38] D. A. Close and P. A. Johnston , “Miniaturization and Characterization of Patient Derived Hepatocellular Carcinoma Tumor Organoid Cultures for Cancer Drug Discovery Applications,” SLAS Discovery 30 (2025): 100201, 10.1016/j.slasd.2024.100201. 39662672 PMC12366521

[39] J. Giron‐Michel , M. Padelli , E. Oberlin , H. Guenou , and J.‐C. Duclos‐Vallée , “State‐of‐the‐Art Liver Cancer Organoids: Modeling Cancer Stem Cell Heterogeneity for Personalized Treatment,” Biodrugs 39, no. 2 (2025): 237–260, 10.1007/s40259-024-00702-0. 39826071 PMC11906529

[40] R. Igarashi , M. Oda , R. Okada , et al., “Generation of human Adult Hepatocyte Organoids With Metabolic Functions,” Nature 641, no. 8065 (2025): 1248–1257, 10.1038/s41586-025-08861-y. 40240606

[41] F. Wu , D. Wu , Y. Ren , et al., “Generation of Hepatobiliary Organoids From human Induced Pluripotent Stem Cells,” Journal of Hepatology 70, no. 6 (2019): 1145–1158, 10.1016/j.jhep.2018.12.028. 30630011

[42] S. P. Harrison , R. Siller , Y. Tanaka , et al., “Scalable Production of Tissue‐Like Vascularized Liver Organoids From human PSCs,” Experimental & Molecular Medicine 55, no. 9 (2023): 2005–2024, 10.1038/s12276-023-01074-1. 37653039 PMC10545717

[43] X.‐C. Sun , D. Kong , J. Zhao , K. N. Faber , Q. Xia , and K. He , “Liver Organoids: Established Tools for Disease Modeling and Drug Development,” Hepatology Communications 7, no. 4 (2023): 0105, 10.1097/HC9.0000000000000105. PMC10043560 36972388

[44] L. Zhu , S. Liu , Z. Wang , et al., “Modeling Hepatic Steatosis With human Adult Stem Cell‐derived Liver Organoids,” Iscience 28, no. 5 (2025): 112344, 10.1016/j.isci.2025.112344. 40276762 PMC12019286

[45] H. Kim and H.‐J. Park , “Current hPSC‐derived Liver Organoids for Toxicity Testing: Cytochrome P450 Enzymes and Drug Metabolism,” Toxicological Research 41, no. 2 (2025): 105–121, 10.1007/s43188-024-00275-8. 40013078 PMC11850699

[46] S. Salas‐Silva , Y. Kim , T. H. Kim , et al., “Human Chemically‐derived Hepatic Progenitors (hCdHs) as a Source of Liver Organoid Generation: Application in Regenerative Medicine, Disease Modeling, and Toxicology Testing,” Biomaterials 303 (2023): 122360, 10.1016/j.biomaterials.2023.122360. 38465578

[47] W. L. Septiana , A. Noviantari , and R. D. Antarianto , “Induced Pluripotent Stem Cells (Ipscs) Based Liver Organoid: The Benefits and Challenges,” Cellular Physiology and Biochemistry : International Journal of Experimental Cellular Physiology, Biochemistry, and Pharmacology 57, no. 5 (2023), 345–359, https://api.semanticscholar.org/CorpusID. 37767740 10.33594/000000662

[48] C. Raggi , S. Selleri , M. M'Callum , and M. Paganelli , “Generation of Complex Syngeneic Liver Organoids From Induced Pluripotent Stem Cells to Model Human Liver Pathophysiology,” Current Protocols 2, no. 3 (2022): 389, 10.1002/cpz1.389. 35263041

[49] S. S. Ng , K. Saeb‐Parsy , S. J. I. Blackford , et al., “Human iPS Derived Progenitors Bioengineered Into Liver Organoids Using an Inverted Colloidal Crystal Poly (ethylene glycol) Scaffold,” Biomaterials 182 (2018): 299–311, 10.1016/j.biomaterials.2018.07.043. 30149262 PMC6131727

[50] R. Nguyen , S. Da Won Bae , L. Qiao , and J. George , “Developing Liver Organoids From Induced Pluripotent Stem Cells (iPSCs): An Alternative Source of Organoid Generation for Liver Cancer Research,” Cancer Letters 508 (2021): 13–17, 10.1016/j.canlet.2021.03.017. 33771683

[51] S. J. Mun , Y.‐H. Hong , Y. Shin , et al., “Efficient and Reproducible Generation of human Induced Pluripotent Stem Cell‐derived Expandable Liver Organoids for Disease Modeling,” Scientific Reports 13, no. 1 (2023): 22935, 10.1038/s41598-023-50250-w. 38129682 PMC10739970

[52] H. J. Kim , G. Kim , K. Y. Chi , and J.‐H. Kim , “ *In Vitro* Generation of Luminal Vasculature in Liver Organoids: From Basic Vascular Biology to Vascularized Hepatic Organoids,” International Journal of Stem Cells 16, no. 1 (2023): 1–15, 10.15283/ijsc22154. 36310029 PMC9978835

[53] Y. Yoon , S. C. Gong , M. Y. Kim , et al., “Generation of Fibrotic Liver Organoids Using Hepatocytes, Primary Liver Sinusoidal Endothelial Cells, Hepatic Stellate Cells, and Macrophages,” Cells 12, no. 21 (2023): 2514, 10.3390/cells12212514. 37947592 PMC10647544

[54] Y. Liu , Y. Zhou , J. Ahodantin , et al., “Generation and Characterization of Mature Hepatocyte Organoids for Liver Metabolic Studies,” Journal of Cell Science 137, no. 10 (2024): jcs261961, 10.1242/jcs.261961. 38700490 PMC11166457

[55] J.‐H. Kim , S. J. Mun , J.‐H. Kim , M. J. Son , and S.‐Y. Kim , “Integrative Analysis of Single‐cell RNA‐seq and ATAC‐seq Reveals Heterogeneity of Induced Pluripotent Stem Cell‐derived Hepatic Organoids,” Iscience 26, no. 9 (2023): 107675, 10.1016/j.isci.2023.107675. 37680467 PMC10481365

[56] J. H. Heo , D. Kang , S. J. Seo , and Y. Jin , “Engineering the Extracellular Matrix for Organoid Culture,” International Journal of Stem Cells 15, no. 1 (2022): 60–69, 10.15283/ijsc21190. 35220292 PMC8889330

[57] D. Kim , H. Lim , J. Youn , T.‐E. Park , and D. S. Kim , “Scalable Production of Uniform and Mature Organoids in a 3D Geometrically‐engineered Permeable Membrane,” Nature Communications 15, no. 1 (2024): 9420, 10.1038/s41467-024-53073-z. PMC11528013 39482314

[58] A. Myszczyszyn , A. Muench , V. Lehmann , et al., “A Hollow fiber Membrane‐based Liver Organoid‐on‐a‐chip Model for Examining Drug Metabolism and Transport,” Biofabrication 17, no. 2 (2025): 025035, 10.1088/1758-5090/adc3ce. 40117762

[59] A. Hess , S. D. Gentile , A. Ben Saad , et al., “Single‐cell Transcriptomics Stratifies Organoid Models of Metabolic Dysfunction‐associated Steatotic Liver Disease,” The EMBO Journal 42, no. 24 (2023): EMBJ2023113898, 10.15252/embj.2023113898. PMC10711666 37962490

[60] H. Yang , J. Cheng , H. Zhuang , et al., “Pharmacogenomic Profiling of Intra‐tumor Heterogeneity Using a Large Organoid Biobank of Liver Cancer,” Cancer Cell 42, no. 4 (2024): 535–551, 10.1016/j.ccell.2024.03.004. 38593780

[61] S. Y. Choi , T. H. Kim , M. J. Kim , et al., “Validating Well‐Functioning Hepatic Organoids for Toxicity Evaluation,” Toxics 12, no. 5 (2024): 371, 10.3390/toxics12050371. 38787150 PMC11126009

[62] S. Gong , K. He , Y. Liu , et al., “Scalable Matrigel‐Free Suspension Culture for Generating High‐Quality Human Liver Ductal Organoids,” Cell Proliferation 58, no. 9 (2025): 70033, 10.1111/cpr.70033. PMC12414634 40166955

[63] H.‐R. Moon , S. J. Mun , T. H. Kim , et al., “Guidelines for Manufacturing and Application of Organoids: Liver,” International Journal of Stem Cells 17, no. 2 (2024): 120–129, 10.15283/ijsc24044. 38773747 PMC11170117

[64] L. Broutier , G. Mastrogiovanni , M. M. Verstegen , et al., “Human Primary Liver Cancer–derived Organoid Cultures for Disease Modeling and Drug Screening,” Nature Medicine 23, no. 12 (2017): 1424–1435, 10.1038/nm.4438. PMC5722201 29131160

[65] S. Ji , L. Feng , Z. Fu , et al., “Pharmaco‐proteogenomic Characterization of Liver Cancer Organoids for Precision Oncology,” Science Translational Medicine no. 706 (2023): adg3358, 10.1126/scitranslmed.adg3358. PMC10949980 37494474

[66] S. Vivarelli , S. Candido , G. Caruso , L. Falzone , and M. Libra , “Patient‐Derived Tumor Organoids for Drug Repositioning in Cancer Care: A Promising Approach in the Era of Tailored Treatment,” Cancers 12, no. 12 (2020): 3636, 10.3390/cancers12123636. 33291603 PMC7761978

[67] T. Ishizaki , Y. Takeuchi , K. Ishibashi , N. Gotoh , E. Hirata , and K. Kuroda , “Cryopreservation of Tissues by Slow‐freezing Using an Emerging Zwitterionic Cryoprotectant,” Scientific Reports 13, no. 1 (2023): 37, 10.1038/s41598-022-23913-3. 36593263 PMC9807565

[68] M. Kaneko , N. Takizawa , T. Wakabayashi , H. Kaneoka , and A. Ito , “Amphiphilic Phospholipid Polymers as a Cryoprotectant for Vitrification and Nanowarming of Rat Livers,” Journal of Bioscience and Bioengineering 139, no. 1 (2025): 70–78, 10.1016/j.jbiosc.2024.10.003. 39455294

[69] O. Rogulska , J. Havelkova , and Y. Petrenko , “Cryopreservation of Organoids,” Cryoletters 44, no. 2 (2023): 65–75, 10.54680/fr23210110112. 37883156

[70] A. Sharma , C. Y. Lee , B.‐E. Namsrai , et al., “Correction: Cryopreservation of Whole Rat Livers by Vitrification and Nanowarming,” Annals of Biomedical Engineering 51, no. 3 (2023): 578–578, 10.1007/s10439-022-03113-w. 36445557

[71] S. Ramesh , J. S. Rao , B.‐E. Namsrai , et al., “Vitrification and Rapid Rewarming of Precision‐cut Liver Slices for Pharmacological and Biomedical Research,” Bioengineering & Translational Medicine 11 (2024): 70045, 10.1101/2024.12.08.627273. PMC12821208 41573366

[72] K. Ziani , L. Saenz‐del‐Burgo , J. L. Pedraz , and J. Ciriza , “Advances in Cryopreservation Strategies for 3D Biofabricated Constructs: From Hydrogels to Bioprinted Tissues,” International Journal of Molecular Sciences 26, no. 14 (2025): 6908, 10.3390/ijms26146908. 40725153 PMC12295925

[73] W. He , Q. Sun , D. Ge , Y. Liu , and B. Sun , “Application of Nanomaterials in Organoid Culture and Cryopreservation,” Nanoscale Advances 7, no. 23 (2025): 7483–7503, 10.1039/d5na00534e. 41210600 PMC12591057

[74] S. J. Mun , Y.‐H. Hong , H.‐S. Ahn , J.‐S. Ryu , K.‐S. Chung , and M. J. Son , “Long‐Term Expansion of Functional Human Pluripotent Stem Cell‐Derived Hepatic Organoids,” International Journal of Stem Cells 13, no. 2 (2020): 279–286, 10.15283/ijsc20060. 32323516 PMC7378903

[75] S. Altmaier , I. Meiser , E. Lemesre , et al., “Human iPSC‐derived Hepatocytes in 2D and 3D Suspension Culture for Cryopreservation and in Vitro Toxicity Studies,” Reproductive Toxicology (Elmsford, N.Y.) 111 (2022): 68–80, 10.1016/j.reprotox.2022.05.005. 35598806

[76] C. M. Gamboa , Y. Wang , H. Xu , K. Kalemba , F. E. Wondisford , and H. E. Sabaawy , “Optimized 3D Culture of Hepatic Cells for Liver Organoid Metabolic Assays,” Cells 10, no. 12 (2021): 3280, 10.3390/cells10123280. 34943788 PMC8699701

[77] M. Tong and S. Ma , “Protocols to Culture and Harvest Hepatic Tumor Organoids for Metabolic Assays,” STAR Protocols 3, no. 3 (2022): 101597, 10.1016/j.xpro.2022.101597. 35942348 PMC9356172

[78] D. Pamies , J. Ekert , M.‐G. Zurich , et al., “Recommendations on Fit‐for‐purpose Criteria to Establish Quality Management for Microphysiological Systems and for Monitoring Their Reproducibility,” Stem Cell Reports 19, no. 5 (2024): 604–617, 10.1016/j.stemcr.2024.03.009. 38670111 PMC11103889

[79] E. Rocco , M. Fumagalli , M. Monturano , et al., “Standard Operating Procedures for Biobank in Oncology,” Frontiers in Molecular Biosciences 9 (2022): 967310, 10.3389/fmolb.2022.967310. 36090048 PMC9459387

[80] M. Wieser , S. Burger , R. Ertl , S. Kummer , M. Stargardt , and I. Walter , “Example for Process Validation in Biobanking: Fit for Purpose Testing of a Cryopreservation Method Without Isopentane,” Frontiers in Molecular Biosciences 9 (2022): 876670, 10.3389/fmolb.2022.876670. 36250023 PMC9562646

[81] D. A. Obeid , T. A. Mir , A. Alzhrani , et al., “Using Liver Organoids as Models to Study the Pathobiology of Rare Liver Diseases,” Biomedicines 12, no. 2 (2024): 446, 10.3390/biomedicines12020446. 38398048 PMC10887144

[82] M. Urbischek , H. Rannikmae , T. Foets , K. Ravn , M. Hyvönen , and M. de la Roche , “Organoid Culture media Formulated With Growth Factors of Defined Cellular Activity,” Scientific Reports 9, no. 1 (2019): 6193, 10.1038/s41598-019-42604-0. 30996238 PMC6470207

[83] Y. Hu , X. Hu , J. Luo , et al., “Liver Organoid Culture Methods,” Cell & Bioscience 13, no. 1 (2023): 197, 10.1186/s13578-023-01136-x. 37915043 PMC10619312

[84] K. Khosla , L. Zhan , A. Bhati , A. Carley‐Clopton , M. Hagedorn , and J. Bischof , “Characterization of Laser Gold Nanowarming: A Platform for Millimeter‐Scale Cryopreservation,” Langmuir 35, no. 23 (2019): 7364–7375, 10.1021/acs.langmuir.8b03011. 30299961 PMC6536355

[85] M. Gross , A. Dewan , M. Macis , et al., “Decentralized Biobanking Platform for Organoid Research networks,” Frontiers in Blockchain 8 (2025): 1510429, 10.3389/fbloc.2025.1510429.

[86] M. D. Wilkinson , M. Dumontier , I. J. Aalbersberg , et al., “The FAIR Guiding Principles for Scientific Data Management and Stewardship,” Scientific Data 3, no. 1 (2016): 160018, 10.1038/sdata.2016.18. 26978244 PMC4792175

[87] A. Bernier , F. Molnár‐Gábor , B. M. Knoppers , et al., “Reconciling the Biomedical Data Commons and the GDPR: Three Lessons From the EUCAN ELSI collaboratory,” European Journal of Human Genetics 32, no. 1 (2024): 69–76, 10.1038/s41431-023-01403-y. 37322132 PMC10267538

[88] M. I. Ortiz‐Lizcano , E. Arias‐Antunez , Á. Hernández Bravo , M. B. Caminero , T. Rojo Guillen , and S. H. Nam Cha , “Increasing the Security and Traceability of Biological Samples in Biobanks by Blockchain Technology,” Computer Methods and Programs in Biomedicine 231 (2023): 107379, 10.1016/j.cmpb.2023.107379. 36731311

[89] D. Froelicher , J. R. Troncoso‐Pastoriza , J. L. Raisaro , et al., “Truly Privacy‐preserving Federated Analytics for Precision Medicine With Multiparty Homomorphic Encryption,” Nature Communications 12, no. 1 (2021): 5910, 10.1038/s41467-021-25972-y. PMC8505638 34635645

[90] A. Fuhr , A. Kurtz , C. Hiepen , and S. Müller , “Organoids as Miniature Twins—Challenges for Comparability and Need for Data Standardization and Access,” Organoids 1, no. 1 (2022): 28–36, 10.3390/organoids1010003.

[91] S. N. Boers and A. L. Bredenoord , “Consent for Governance in the Ethical Use of Organoids,” Nature Cell Biology 20, no. 6 (2018): 642–645, 10.1038/s41556-018-0112-5. 29784910

[92] A. J. Hood , M. Macis , D. M. Cervantes , et al., “Privacy, Policy, and Profits: Survey of Patient Preferences for Research on De‐Identified Biosamples,” Journal of Empirical Research on Human Research Ethics 20, no. 3 (2025): 149–161, 10.1177/15562646251346940. 40501291

[93] A. Sharma , J. C. Bischof , and E. B. Finger , “Liver Cryopreservation for Regenerative Medicine Applications,” Regenerative Engineering and Translational Medicine 7, no. 1 (2021): 57–65, 10.1007/s40883-019-00131-4.

[94] N. Jalan‐Sakrikar , T. Brevini , R. C. Huebert , and F. Sampaziotis , “Organoids and Regenerative Hepatology,” Hepatology 77, no. 1 (2023): 305–322, 10.1002/hep.32583. 35596930 PMC9676408

[95] M. B. Afonso , V. Marques , S. W. C. van Mil , and C. M. P. Rodrigues , “Human Liver Organoids: From Generation to Applications,” Hepatology 79, no. 6 (2024): 1432–1451, 10.1097/HEP.0000000000000343. 36815360 PMC11095893

[96] G. Dagher , “Quality Matters: International Standards for Biobanking,” Cell Proliferation 55, no. 8 (2022): 13282, 10.1111/cpr.13282. PMC9357355 35709534

[97] D. Pamies , A. Bal‐Price , A. Simeonov , et al., “Good Cell Culture Practice for Stem Cells and Stem‐cell‐derived Models,” Altex ‐ Alternatives to Animal Experimentation 34, no. 1 (2017): 95–132, 10.14573/altex.1607121. 27554434

[98] H. Han , T. Zhan , N. Guo , M. Cui , and Y. Xu , “Cryopreservation of Organoids: Strategies, Innovation, and Future Prospects,” Biotechnology Journal 19, no. 2 (2024): 202300543, 10.1002/biot.202300543. 38403430

[99] L. Coppola , A. Cianflone , A. M. Grimaldi , et al., “Biobanking in Health Care: Evolution and Future Directions,” Journal of Translational Medicine 17, no. 1 (2019): 172, 10.1186/s12967-019-1922-3. 31118074 PMC6532145

[100] A. M. Wong , H. Huang , A. M. Wong , et al., “Patient‐derived Organoids Inform Pharmacogenomic Vulnerabilities in Liver Cancer,” JHEP Reports 7, no. 7 (2025): 101426, 10.1016/j.jhepr.2025.101426. 40584266 PMC12205652

[101] T. Shinozawa , M. Kimura , Y. Cai , et al., “High‐Fidelity Drug‐Induced Liver Injury Screen Using Human Pluripotent Stem Cell–Derived Organoids,” Gastroenterology 160, no. 3 (2021): 831–846, 10.1053/j.gastro.2020.10.002. 33039464 PMC7878295

[102] J. Rao , C. Song , Y. Hao , et al., “Leveraging Patient‐Derived Organoids for Personalized Liver Cancer Treatment,” International Journal of Biological Sciences 20, no. 13 (2024): 5363–5374, 10.7150/ijbs.96317. 39430248 PMC11488587

[103] N. Prior , P. Inacio , and M. Huch , “Liver Organoids: From Basic Research to Therapeutic Applications,” Gut 68, no. 12 (2019): 2228–2237, 10.1136/gutjnl-2019-319256. 31300517 PMC6872443

[104] H. Li , H. Liu , and K. Chen , “Living Biobank‐based Cancer Organoids: Prospects and Challenges in Cancer Research,” Cancer Biology & Medicine 19, no. 7 (2022): 965–982, 10.20892/j.issn.2095-3941.2021.0621. 35856555 PMC9334762

[105] HYBRIDA Consortium. (n.d.) . Operational guidelines regarding organoids and organoid‐related technologies.

[106] Y. Zhu , S. Tang , Q. Yuan , et al., “Integrated Characterization of Hepatobiliary Tumor Organoids Provides a Potential Landscape of Pharmacogenomic Interactions,” Cell Reports Medicine 5, no. 2 (2024): 101375, 10.1016/j.xcrm.2023.101375. 38278146 PMC10897507

[107] S. Akbari , G. G. Sevinç , N. Ersoy , et al., “Robust, Long‐Term Culture of Endoderm‐Derived Hepatic Organoids for Disease Modeling,” Stem Cell Reports 13, no. 4 (2019): 627–641, 10.1016/j.stemcr.2019.08.007. 31522975 PMC6829764

[108] Y. Guan , D. Xu , P. M. Garfin , et al., “Human Hepatic Organoids for the Analysis of human Genetic Diseases,” JCI Insight 2, no. 17 (2017): 94954, 10.1172/jci.insight.94954. 28878125 PMC5621886

[109] X. Wu , D. Jiang , Y. Yang , S. Li , and Q. Ding , “Modeling Drug‐induced Liver Injury and Screening for Anti‐hepatofibrotic Compounds Using human PSC‐derived Organoids,” Cell Regeneration 12, no. 1 (2023): 6, 10.1186/s13619-022-00148-1. 36864321 PMC9981852

[110] W. Senkowski , L. Gall‐Mas , M. M. Falco , et al., “A Platform for Efficient Establishment and Drug‐response Profiling of High‐grade Serous Ovarian Cancer Organoids,” Developmental Cell 58, no. 12 (2023): 1106–1121, 10.1016/j.devcel.2023.04.012. 37148882 PMC10281085

[111] Y. Chen , Y. Liu , S. Chen , et al., “Liver Organoids: A Promising Three‐dimensional Model for Insights and Innovations in Tumor Progression and Precision Medicine of Liver cancer,” Frontiers in Immunology 14 (2023): 1180184, 10.3389/fimmu.2023.1180184. 37334366 PMC10272526

[112] W. Shao , H. Xu , K. Zeng , M. Ye , R. Pei , and K. Wang , “Advances in Liver Organoids: Replicating Hepatic Complexity for Toxicity Assessment and Disease Modeling,” Stem Cell Research & Therapy 16, no. 1 (2025): 27, 10.1186/s13287-025-04139-2. 39865320 PMC11771052

[113] C. C. Bell , D. F. G. Hendriks , S. M. L. Moro , et al., “Characterization of Primary human Hepatocyte Spheroids as a Model System for Drug‐induced Liver Injury, Liver Function and Disease,” Scientific Reports 6, no. 1 (2016): 25187, 10.1038/srep25187. 27143246 PMC4855186

[114] S. Ariño , R. Ferrer‐Lorente , G. Serrano , et al., “Patient‐derived Liver Organoids Recapitulate Liver Epithelial Heterogeneity and Enable Precision Modeling of Alcohol‐related Liver Disease,” Journal of Hepatology 84, no. 1 (2026): 135–149, 10.1016/j.jhep.2025.07.014. 40754225

[115] A. Messina , E. Luce , M. Hussein , and A. Dubart‐Kupperschmitt , “Pluripotent‐Stem‐Cell‐Derived Hepatic Cells: Hepatocytes and Organoids for Liver Therapy and Regeneration,” Cells 9, no. 2 (2020): 420, 10.3390/cells9020420. 32059501 PMC7072243

[116] J. Zhao , Y. Zhi , H. Ren , J. Wang , and Y. Zhao , “Emerging Biotechnologies for Engineering Liver Organoids,” Bioactive Materials 45 (2025): 1–18, 10.1016/j.bioactmat.2024.11.002. 39588483 PMC11585797

[117] J.‐C. Lin , Y.‐L. Liu , W. W.‐W. Hsiao , and C.‐T. Fan , “Integrating Population‐based Biobanks: Catalyst for Advances in Precision Health,” Computational and Structural Biotechnology Journal 24 (2024): 690–698, 10.1016/j.csbj.2024.10.049. 39624826 PMC11609404

